# Exploration of multivariate analysis in microbial coding sequence modeling

**DOI:** 10.1186/1471-2105-13-97

**Published:** 2012-05-14

**Authors:** Tahir Mehmood, Jon Bohlin, Anja Bråthen Kristoffersen, Solve Sæbø, Jonas Warringer, Lars Snipen

**Affiliations:** 1 Biostatistics, Department of Chemistry, Biotechnology and Food Sciences, Norwegian University of Life Sciences, Aas, Norway; 2 EpiCenter, Department of Food Safety and Infection Biology, , Oslo, Norway; 3 Section for Epidemiology, Norwegian Veterinary Institute, Oslo, Norway; 4 Department of Informatics, University of Oslo, Oslo, Norway; 5 Department of Chemistry and Molecular Biology, University of Gothenburg, Gothenburg, Sweden; 6 Center of Integrative Genetics (CIGENE) and Department of animal and aquaculture, Norwegian University of Life Sciences, Aas, Norway

## Abstract

**Background:**

Gene finding is a complicated procedure that encapsulates algorithms for coding sequence modeling, identification of promoter regions, issues concerning overlapping genes and more. In the present study we focus on coding sequence modeling algorithms; that is, algorithms for identification and prediction of the actual coding sequences from genomic DNA. In this respect, we promote a novel multivariate method known as Canonical Powered Partial Least Squares (CPPLS) as an alternative to the commonly used Interpolated Markov model (IMM). Comparisons between the methods were performed on DNA, codon and protein sequences with highly conserved genes taken from several species with different genomic properties.

**Results:**

The multivariate CPPLS approach classified coding sequence substantially better than the commonly used IMM on the same set of sequences. We also found that the use of CPPLS with codon representation gave significantly better classification results than both IMM with protein (*p* < 0.001) and with DNA (*p* < 0.001). Further, although the mean performance was similar, the variation of CPPLS performance on codon representation was significantly smaller than for IMM (*p* < 0.001).

**Conclusions:**

The performance of coding sequence modeling can be substantially improved by using an algorithm based on the multivariate CPPLS method applied to codon or DNA frequencies.

## Background

For each sequenced genome, the basic step of annotation is the prediction of genes. In prokaryotes, an average of over 80% of the genome consists of genes which are mostly protein coding [1], meaning that correct identification of protein coding genes is a key aim in computational biology. A complicating factor is that a fraction of microbial genomes consist of degenerated genes with no remaining functionality [2]. A gene finder must therefore be a rather complex ’engine’ capable of distinguishing real protein-coding genes from DNA sequence regions consisting of degenerated genes, non-coding regions and more. To map genes, gene finders typically identify a set of gene-candidates commonly referred to as open reading frames (ORFs). The number of ORFs found by gene finders is typically large compared to the true number of genes. To reduce the number of ORFs and minimize the false predictions of real protein-coding genes, a gene finder must take into account several genomic properties like the existence of upstream regulatory sequences (ribosomal binding sites, promoter regions, etc.), degree and type of overlap between open reading frames, as well as the content of the coding genes themselves. Considering the above mentioned properties, a gene finding procedure can be sketched as follows: 1) identify all possible ORFs in the genome, 2) score all ORFs by various criteria, e.g. their length, their base composition, their upstream sequence, their overlap with other ORFs, etc. 3) classify ORFs as coding genes or non-coding regions based on the scores achieved in the previous steps.

Although the performance of prokaryotic gene finders is relatively good compared to eukaryotic gene finders [3,4] there is room for improvement. Prokaryotic gene finders have a tendency to be biased towards identifying false positive ORFs [5]. Short genes are difficult to identify correctly [4], and genes in GC rich genomes are challenging to predict accurately [6-8]. It is therefore important that proper algorithms for coding sequence modeling are implemented in gene finders. Algorithms used by gene finders should have the ability to extract sequence parameters from coding sequence modeling of putative genes (often referred to as training), and then classify new genes as ORFs based on similarity to the estimated sequence parameters [9]. Several popular gene finders use models based on some sort of Markov chain methodology to identify ORFs [10-15]. Markov chain based models are ”trained” on a set of sequences (typically nucleotide, protein or codon frequencies) and use the statistical parameters extracted from this training to classify new sequences [16]. The training procedure in Glimmer3 [6], which is a Markov chain-type model, identifies long ORFs from DNA sequences which are used to build the Interpolated Context Model. The Interpolated Context Model (IMM) is then used to classify ORFs in DNA sequences having similar characteristics to the training data sequences. This means that the classification power of gene finders based on training relies heavily on the properties of the sequence data used. Thus, for gene finders, it is important that the sequence data used for training has as many general characteristics of genes as possible, which emphasizes the relevance of procedures that facilitates sequence data for accurate gene prediction. To obtain sequence data that may have such characteristics we turn to pangenomics [17]. The re-sequencing of multiple strains within the same species or phylotype has resulted in the study of microbial pangenomes [17-21]. A pangenome is the collection of genes found in all strains within a population. By considering the set of highly conserved genes within a pangenome, we are close to obtaining a data set consisting of “true” genes since these sequences are highly conserved across many strains and are therefore considerably more reliable than data sets based on genes from one genome sequence only. Thus, we argue that data sets consisting of genes obtained from pangenomic inspired analyses may be an adequate starting point for a general testing and comparison of gene finders. Indeed, we use such sequence data to compare the capabilities of a multivariate coding sequence modeling algorithm using different methodology to that of the Markov chain based coding sequence modeling algorithms. Although multivariate methods (e.g. [22,23]) are extensively applied in other scientific fields only one such method known to the authors has been suggested as a gene finding algorithm [24]. Data sets used for gene finding typically have a large number of variables *p* (usually frequency counts of oligonucleotide like codons) in comparison to the number of ORFs *n*. As a consequence we have to deal with the unbalanced *p* >*n* situation, making it hard to classify ORFs since unique estimates cannot be found. Multivariate tools like Partial Least Square (PLS) regression are widely used to address unbalanced *p* >*n* problems [25]. A recent advancement to the PLS regression scheme combines a novel data compression method, canonical correlation analysis (CCA), to additionally estimate latent variables enhancing classification in regression type problems even further. This method has been termed Canonical Powered Partial Least Squares (CPPLS) [22] and we explore the performance on the modeling of coding genes.

## Method

### Approach

#### Gene modeling data

The genomic data which was used to train the coding sequence modeling algorithms was divided into two groups. One group, termed ‘Positives’, contained ORFs considered to be real genes. The other group, termed ‘Negatives’, consisted of ORFs known to be non-coding, *i.e.* sequences recognized as non-genes. We only considered protein coding genes in this study.

##### Positives

To assure that the data set representing coding genes was as reliable as possible, we applied an approach, outlined below, based on RefSeq [26] annotated genes from multiple strains ( http://www.ncbi.nlm.nih.gov/RefSeq/). RefSeq genes are considered to be comprehensive, non-redundant and well-annotated. We studied 12 prokaryote species having at least 4 completed genomes with RefSeq-annotations available (see Table 1). Genomes that were sequenced twice were excluded. All the genomes of these species were downloaded from NCBI ( http://www.ncbi.nlm.nih.gov/genome), together with their RefSeq-annotated genes ( http://www.ncbi.nlm.nih.gov/RefSeq/). The lists of RefSeq genes for all genomes within each species were compared by an all-against-all reciprocal megaBLAST [27] search. For any two ORFs, a pairwise distance was computed as follows: If *s*(*i*;*j*) is the bitscore of the alignment between query sequence *i* and database sequence *j*, the distance between them is given by:

(1) $$d(i,j)=1-\frac{s(i;j)+s(j;i)}{s(i;i)+s(j;j)}$$

**Table 1:** An overview of the species

**Species**	**Group**	**Number of genomes**	**GC-content**
*Acinetobacter baumannii*	Gammaproteobacteria	6	0.39
*Bacillus cereus*	Firmicutes	9	0.36
*Bifidobacterium longum*	Actinobacteria	4	0.60
*Chlamydia trachomatis*	Chlamydiae/Verrucomicrobia	6	0.41
*Escherichia coli*	Gammaproteobacteria	25	0.50
*Mycobacterium tuberculosis*	Actinobacteria	5	0.65
*Pseudomonas putida*	Gammaproteobacteria	4	0.62
*Rhodopseudomonas palustris*	Alphaproteobacteria	6	0.65
*Staphylococcus aureus*	Firmicutes	15	0.33
*Streptococcus pneumoniae*	Firmicutes	14	0.40
*Streptococcus pyogenes*	Firmicutes	13	0.37
*Sulfolobus islandicus*	Crenarchaeota	7	0.35

**Figure 1:**
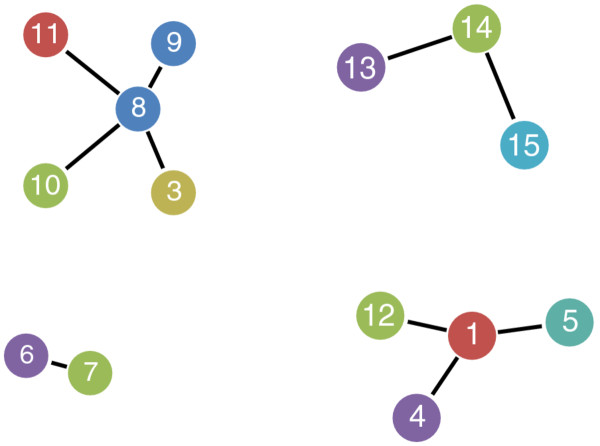
**Visualization of undirected graph.** The clusters of highly conserved ORFs are presented, based on a very small subset taken from *Acinetobacter baumannii*. Nodes represent genes, and identical color means genes from the same genome. The numbers are just identifiers within each genome. First we discard the clusters having less than 3 genes, i.e. red:6-yellow:7. Next, the medoide gene from each remaining cluster forms the set of Positives.

An overview of the species used in the current study along with respective group, number of genomes and GC-content.

where *d*(*i*,*j*) always gives a value between 0 and 1. Next, all ORFs were represented as nodes in an undirected graph, with edges added between two nodes if the corresponding distance *d*(*i*,*j*) between them was below or equal to some threshold *t* that designates sequence similarity. Hence, we considered two ORFs to be connected if they were sufficiently similar according to a specified threshold value *t*. If multiple ORFs fulfill this similarity criterion a graph will form consisting of many nodes (ORFs). Such a graph will form clusters of connected nodes. Clusters with nodes designating ORFs from the genomes of multiple strains are more likely to be real coding genes since they are conserved across several genomes. A highly conserved ORF (HCO) is therefore represented as a graph with nodes from the genomes of all respective strains within a species. For each HCO cluster, the node (ORF) with the smallest sum of distances, as measured using the weighted edges to all other nodes (ORFs) in the same cluster, was extracted. Such nodes are referred to as medoids. The medoide thus represents the whole HCO cluster. The same procedure is subsequently applied repeatedly generating a list of HCOs for each species. The list of HCOs for each species contains our candidate genes and we designate that set as Positives. For illustration purposes Figure 1 shows a visualized graph for a very small data subset taken from *Acinetobacter baumannii*.

##### Negatives

Algorithms involved in coding sequence modeling must separate sequences that are genes and sequences that are not genes. Sequences that are not genes are designated Negatives. The set of Negatives will further enable classifying sequences as coding or non-coding genes. Negatives are considerably harder to identify than Positives since prokaryotic genomes are densely covered with genes. Even if a sequence is not among our HCOs it may very well be a coding gene, or at least part of a coding gene. However, the reading frame is an indispensable concept with respect to coding sequences, as elaborated by [28], and due to different selection pressure in-frame and out-of-frame sequences are evaluated differently and form completely separate clusters [24]. Consequently, we consider the out-of-frame interior from the set of Positives as Negatives in the current study. This implies that no Positive has a complete overlap with another Positive. It is, however, typically accepted that functional genes in prokaryotes can overlap over short stretches [29]. Hence, a small fraction of our Negatives may actually be part of a gene, making Negatives difficult to classify correctly. There are always 5 out-of-frame reading frames, and all are considered as Negatives, *i.e.* for each Positive we have 5 Negatives. Sequences designated as Negatives will hence not have a proper start and stop codon, but are likely to contain spurious stop codons since they are out-of-frame. In order to use this approach, we therefore eliminated the first start- and all stop-codons from every sequence labeled as either Negative or Positive.

##### Data splitting and cross-validation

For each species, the sets containing Positives and Negatives were randomly divided into 10 equally sized subsets. One of these subsets was used as test data while the other 9 subsets were used as training data. The procedure was repeated in a 10-fold cross-validation.

#### ORF sequence representation

Genes can be represented as DNA sequences, codon sequences or protein (amino acid) sequences. We describe all representations below with respect to coding sequence modeling.

##### DNA sequences

The DNA alphabet consists of 4 symbols; but the reading frame concept must also be taken into consideration. Hence, the bases we observe in codon positions 1, 2 and 3 must be considered separately, otherwise it is impossible to distinguish in-frame from out-of-frame sequences. Markov chain models therefore need three separate sets of transition probabilities, each set corresponding to the target symbol in reading frame 1, 2 or 3. The pretext, *i.e.* the subsequence a Markov chain model is conditioned upon, consists of all preceding *k* symbols regardless of which reading frame is considered. A Markov chain model will therefore traverse a DNA sequence, nucleotide by nucleotide, constantly consulting transition probabilities from all reading frames. Such is the case for GeneMark [11] and GLIMMER [13]. From this perspective, the DNA alphabet of coding sequences has 4 ∗ 3 = 12 and not 4 symbols.

##### Codon sequences

Each protein coding gene may also be represented by its codon alphabet. The codons consist of three consecutive nucleotides and code for amino acids, thereby giving 64 possible combinations. Ignoring the 3 exclusive stop codons, 61 symbols are free to code for amino acids. Since there are only 20 different standard amino acids, the codon alphabet is redundant. In other words, some codons code for the same amino acid. Hence, some codons are synonymous while others are non-synonymous. In fact, the redundancy of the codon alphabet allows organisms and genes to prefer specific codons coding for specific amino acids. This is typically known as codon bias [30]. Although the codon alphabet, with its 61 symbols, provides more resolution than the DNA and protein alphabets, the added information can be a computational challenge.

##### Protein sequences

Due to the redundancy of the codon alphabet gene comparisons may often be more successful using protein sequences. Since different codons can code for the same amino acid, DNA sequences representing homologue genes may be very different in terms of base composition and therefore hard to detect using DNA based search engines. In such cases, using protein sequences instead of DNA sequences may give better results since there is no redundancy. Protein sequences are expected to be highly conserved by purifying selection, in contrast to the more variable DNA sequences [24].

### Algorithm

#### Classification of coding sequence

The methods used to classify genes were Interpolated Markov model (IMM) [13] and Canonical Powered PLS (CPPLS) [22]. Both models need to be trained and from the training data set of *n* sequences we create a *n* × 1 numeric response vector **y** containing the value 1 if the respective sequence is from the Positive set and -1 if the respective sequence is from the Negative set.

##### Interpolated Markov models (IMM)

Markov chain models are widely used to detect patterns in biological sequences. Unfortunately, these models are hampered by the necessity to find the appropriate order of the Markov chain. A higher order Markov chain model has more parameters and therefore less bias since it is capable of describing more accurately the real probabilities behind the observed sequences. However, for a fixed size data set the information per parameter is less, resulting in estimators with increased variance [31]. Thus, the improvement obtained due to less bias may be lost to the increased variance. A fifth order Markov chain model is employed by GeneMark, while the gene finding algorithm in Glimmer is based on the interpolated Markov model (IMM). The latter model (IMM) estimates several chains with different orders, of which the separate scores are subsequently combined into one, making it a more general approach than the prior 5^*th*^ order model. Since we are comparing coding sequence modeling algorithms we use the IMM approach used by the Glimmer software [13]. This means that the final probability of a symbol is a linear combination of several Markov chain models from order *k* = 0 up to some upper limit *k* = *K*, where the Markov chain transition probabilities from various orders are weighted based on the size and information content of the training data. Some additional effort is required to estimate these weights since there is no closed form solution for the maximum of the likelihood function. The Expectation Maximization (EM) algorithm [32] is applied iteratively to find local optimum solutions which are consequently applied to optimize the weights used in the linear interpolation. From the training data two interpolated Markov chain models are fitted, one for Positives (+1) and the other for Negatives (-1). Thus, for both Positives and Negatives we need to estimate the transition probability matrices $T_{1}^{+},\ldots ,T_{K}^{+}$ along with the weights used in the interpolation procedure. Then, for each sequence from the test data the posterior log-probability scores for the Positive and Negative models are computed using the estimated transition probability matrices and weights. Finally, each test set of sequences is assigned to the class (+1 or -1) depending on the log-probability score. In an approach like this, the upper model order *K* must be restricted due to space and computation time limitations. For the codon alphabet, having 61 symbols, even a second order model (*K = 2*) includes 6^13^ = 226981 transition probabilities, and is therefore computationally very slow. Also, a training set of considerable size is required to estimate all probabilities with reasonable variance. The addition of pseudo counts is considered useful method to stabilize the estimates of a Markov chain model [33]. We have chosen to use this as well, but in a very careful way. If we have *m* observations (transitions/initiations) in our data set, we add $\sqrt[{4}]{m}$ pseudo counts as well, all having probabilities given by a 0-order Markov chain model for the Positives or Negatives, respectively.

##### Canonical Powered PLS (CPPLS)

From the training data set of *n* sequences, together with response *y*, the predictor *n* × *p* matrix ***X*** is formed by word frequencies for each sequence from the training data. A word is a fixed length consecutive segment from the sequence. Since the amount of information required for a *k*^*th*^ order Markov chain model corresponds to *k* + 1 word frequencies, all words of length from 1 up to *K* + 1 were included to make this approach comparable. The association between ***y*** and the predictor matrix ***X*** is assumed to be explained by the linear model, *i.e*.

(2) E(y)=Xβ

***β*** are *p* regression coefficients relating every word frequency to the class status (+1 or -1). This results in a ’large *p* and small *n*’ situation, where ordinary least squares type methods provide poor solutions. The PLS method can estimate the regression coefficients for such a case using an iterative procedure described in [25]. There are many algorithms in the PLS-family, and for classification purposes we use the CPPLS method [22]. Thus, from the training data we estimate the regression vector ***β*** describing the contrast between Positives and Negatives. For a given test sequence, the corresponding word frequency 1 × *p* vector ***x*** is computed. Based on the CPPLS estimated regression coefficients $\hat{\beta }$ a score is predicted by $\hat{y}=X\hat{\beta }$ classified as +1 or -1, that is as Negative or Positive [9].

#### Model sizes

In general, the performance of a classifier is linked to the number of parameters being estimated. For the Markov chain model, this means the number of transition probabilities and weights, while for the PLS-approach it means the number of regression coefficients. The optimal model complexity, which is measured by the number of free parameters, is always a trade-off between bias and variance [34]. Since comparisons are carried out between different methods and sequence representations, there should be a comparable number of parameters. Table 2 presents the number of transition probabilities to be estimated for all three sequence representations using interpolated Markov chain models of different orders. It appears that for a reasonably fair comparison with the CPPLS method, the interpolated Markov chain model should be of order 4 for DNA, order 2 for protein and order 1 for codon sequences. It is important to recall that the number of transition probabilities required for a *k*^*th*^ order Markov chain model corresponds to *k* + 1 word frequencies. Hence, for the CPPLS method frequencies of 4-words, 3-words and 2-words are used for codon, protein, and DNA sequences, respectively.

**Table 2:** The number of probabilities to be estimated in an IMM

**Sequence type**	***k = *0**	***k = *1**	***k = *2**	***k = *3**	***k = *4**	***k = *5**
DNA	12	60	252	1020	4092	16380
Protein	20	420	8420	168420	3368420	67368420
Codon	61	3782	230763	14076604	858672905	52379047266

The columns represent the number of transition probabilities to be estimated with an Interpolated Markov model from *k = 0* to *k = 5*, while the rows designate the different sequence types (DNA, codon and protein). The number of probabilities in a *k*^*th*^ order IMM corresponds to the number of regression coefficients for the *k* + 1 word frequencies in the CPPLS method.

#### Mixed effect model

The main objective of the study is to make comparisons of methods (CPPLS vs. IMM) and sequence representations (codon vs. protein vs. DNA) on the ability to classify coding sequences. The study has been conducted on genomes from many different species, and in order to present all results in a single analysis, we have adopted an analysis of variance (ANOVA) approach. We were primarily interested in how the choice of method and sequence representation affected the classification performance (outcome), and the (random) variability in results between species should be considered as random ’noise’ in the analysis. This was accomplished by the use of a mixed-effect ANOVA model, where the fixed effects on performance are the focus of our attention (method and sequence representation) and a random effect of species is included to deal with variation between species.

The performance is defined as the percentage of correctly classified ORFs in a test data set using 10-fold cross validation. ANOVA analyses assume constant performance variance at different levels of the fixed effects, which was originally not the case in our data set. To stabilize the variance, the original performance *y* (percentages) was transformed to *z* as $z=\sin^{-1}\sqrt{y/100}$.

We fitted the following mixed effect model

(3) $$z_{i,j,k}=\mu +\alpha_{i}+\beta_{j}+{\left(\alpha \beta \right)}_{i,j}+s_{k}+e_{i,j,k}$$

where the outcome *z*_*i*,*j*,*k*_ is the observed transformed performance, *μ* is the overall expected transformed performance level, *α*_*i*_ is the fixed effect of method *i* = 1, 2, *β*_*j*_ is the fixed effect of sequence representation *j* = 1,2,3, (*αβ*)_*i*,*j*_ is the interaction term combining method *i* and sequence representation *j*, *s*_*k*_ is the random effect of species *k* = 1, …, 12 and *e*_*i*,*j*,*k*_ is the residual variation. As part of the model assumptions in a standard ANOVA we used normal distributed error terms $s_{k}∼N(0,\sigma_{s}^{2})$ and $e_{i,j,k}∼N(0,\sigma_{e}^{2})$.

## Results and discussion

### Data sets

Even if the RefSeq database is curated, there may still be errors. In order to eliminate uncertain sequences we only considered those which were conserved across all genomes within each species. Additional file 1: Figure S1 shows how the number of gene clusters grows by the choice of threshold *t*, which represents the similarity between sequences inside a cluster. In our analysis we have chosen to use *t*=0.3, meaning that clusters will contain sequences that are roughly 100*%* (1 − *t*) = 70*%* similar. For each such cluster having members from all genomes, we allocate the medoide sequence to the set of Positives for the corresponding species. As seen from Additional file 1: Figure S1, this results in a rather large number of Positives for all species and we are assured that these sequences are coding genes. So instead of taking all HCOs at *t* = 0.3, if a species has more than 400 HCO’s, we sampled 400 sequences at random as Positives. We have chosen to use as Negatives sequences that constitute the out-of-frame interior of the Positives. The reason for this is actually straightforward; coding genes predominantly cover prokaryotic genomes therefore the intergenic regions are few and small. For instance, the RefSeq annotated genes cover, on average, more than 92% of the genomes in this study. On the other hand, annotations of genes with large overlaps are few in number; therefore we assume that if there is some region where we know there is a coding gene, there will be a small chance that any other coding gene is present in the same region. Thus, we presume that sequences from the out-of-frame interior of the Positives are the types of sequences that have the same base compositional properties as the majority of non-coding ORFs (i.e. Negatives). We also eliminated the first codon (start) as well as all stop-codons from both Positives and Negatives, in order to make the classifications based on the content and not the endpoints.

### Coding sequence recognition

**Figure 2:**
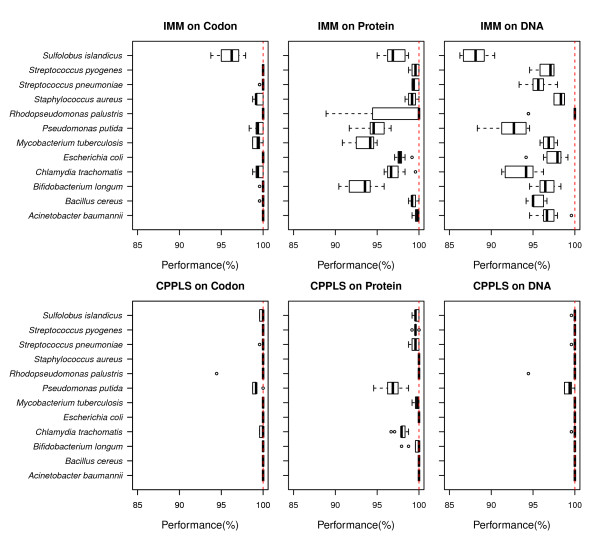
**Performance on test data.** The box and whisker plots show the distributions of performance (% correct classified) on test data for each species, by using IMM (upper panels) or CPPLS (lower panels) on ORFs represented as codon, protein or DNA sequences. The dotted red line indicates the maximum possible performance (100%). For most of the species, CPPLS on Codon sequence performance is 100 (%).

In Figure 2 we show the distributions of performance for each species by applying both the IMM and CPPLS methods on codon, protein and DNA sequences. The difference between the IMM method (upper panels) and the CPPLS method (lower panels) is the most striking result. It can be seen that the codon representation (leftmost panels) appears to be better than protein and DNA, especially for the IMM-approach. We observe non-constant variance of performance over different levels, for instance, an F-test indicates that the variation observed using CPPLS with codon representation was significantly smaller than the corresponding variance for IMM (*p* < 0.001) based on the original performance measure. To make a more formal test, we used a mixed interaction effect ANOVA-type model (see Method) with results presented in Table 3 based on transformed performance. The analysis supports that significant variation among levels of methods (*p* < 0.001), sequences (*p* < 0.001) and method sequence interaction (*p* < 0.001). A Tukey test [35] with adjusted p-values for multiple comparisons, was carried out to compare the difference of means of (transformed) performance between methods and sequence representations. We found that CPPLS performed, in general, better than IMM (*p* < 0.001), while codons were better sequence representations than both protein and DNA (*p* < 0.001). No difference was found between the latter two sequence representations. Further, testing for method and sequence interaction, we found that CPPLS with codon representation performed significantly better than IMM with protein (*p* < 0.001) and with DNA (*p* < 0.001) representations. Mean performance of IMM with codon representation was similar to CPPLS with codon representation, but variation of results were significantly lower for CPPLS (*p* < 0.001) indicating superior performance. The estimated standard deviation of transformed performance due to random effect of species was ${\hat{\sigma }}_{s}=0.077$, which is bigger than the general error term ( ${\hat{\sigma }}_{e}=0.049$). This indicates that performance varies a lot between species (Table 3). In general, the average performance for both the IMM and CPPLS algorithms is very good. Even the worst combination, using IMM on DNA data, has more than 95% correct classifications (both Positives and Negatives) in the majority of the performed tests. Thus, both the IMM and CPPLS methods support the notion that the Positive and Negative sequences have a base composition more intrinsically similar to each other and, therefore, that our division of sequences into these two categories is meaningful. The high performance is largely an effect of our strict choice of threshold *t* when selecting Positives. We only included as Positives the highly conserved genes, and it is quite likely that these genes have more in common than less conserved genes. We also tried more lenient thresholds, giving larger and more heterogeneous sets of Positives (and Negatives), subsequently resulting in a drop in overall performance. However, the differences between methods and sequence representations found for subset of *t* = 0.3 hold throughout.

**Table 3:** Analysis of variance for a mixed effect design in coding sequence modeling

	**Sum of squares**	**DF**	**Mean squares**	**F-value**	**p-value**	
Method	0.19	1	0.189	92.92	*p* < 0.001	
Sequence	0.08	2	0.040	19.53	*p* < 0.001	
Method:Sequence	0.08	2	0.038	18.66	*p* < 0.001	
Species	0.10	11	0.009	4.45	*p* < 0.001	
Residual	0.11	55	0.01			

Analysis of variance for transformed performance (see Method section) as an effect of Method (IMM or CPPLS), Sequence (sequence representation DNA, protein, codon) and their interaction Method:Sequence. The estimated standard deviation of the random effect of Species is ${\hat{\sigma }}_{s}$*= 0.077* and for the Residual ${\hat{\sigma }}_{e}$* = 0.049*.

**Figure 3:**
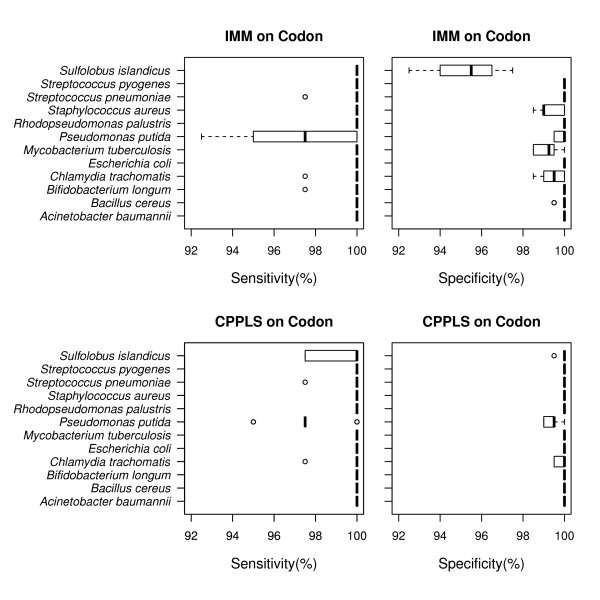
**Sensitivity and specificity.** The distributions of sensitivity and specificity for each species, by using IMM and CPPLS on codon sequences only. Sensitivity is defined as the ability to detect Positives and specificity as the ability to detect Negatives and both are presented in (%).

**Figure 4:**
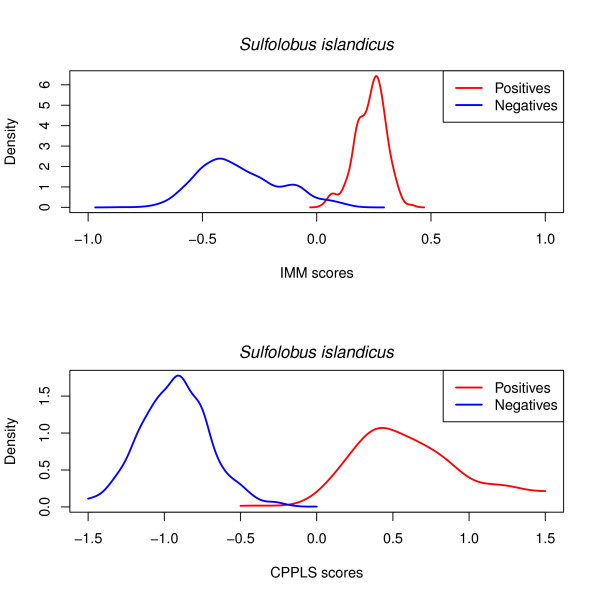
**IMM and CPPLS scores.** For *Sulfolobus islandicus*, the density of the IMM scores and CPPLS scores are plotted. For each test sequence IMM score is computed as the difference of Positive log-probability and Negative log-probability, and CPPLS scores are simply the fitted values.

It should also be noted that the archaeon *Sulfolobus islandicus* gives a notable drop in performance for the IMM, but less so for the CPPLS. This is possibly explained by a difference in variance in the sets of Positives and Negatives. We expect Positive sequences (coding genes) to be more homogenous than Negatives (non-coding ORFs). In any genome, the number of non-coding ORFs is many magnitudes larger than the number of coding genes and since these non-coding orfs are regarded as Negatives the variance in this set is considerably larger than the Positives set. It is therefore reasonable to expect this difference in homogeneity between the Positives and Negatives. When fitting Markov chain models to the Positives and the Negatives, we end up describing the ’average’ of both classes without taking the heterogeneity of their respective variances into account. Hence, for IMM, information about within-class heterogeneity and class size is lost. For CPPLS the regression coefficient estimates are affected by both the average and the variance in word-frequencies, as well as the number of sequences within each class. To illustrate this effect, sensitivity (the ability to identify Positives) and specificity (the ability to identify Negatives) were computed for both methods using codon frequencies (Figure 3). Sensitivity is on average the same for both methods, but CPPLS exhibited a stronger ability to identify Negatives. For further understanding why a multivariate approach like CPPLS outperforms IMM, we have focused on the results for *Sulfolobus islandicus*, with codon representation. Figure 4 presents the density of the IMM scores and CPPLS scores. For each test sequence, the IMM score is computed as the difference of Positive log-probability and Negative log-probability, and CPPLS scores are simply the fitted values. It is clear from Figure 4 that the area of overlap between the red and blue density is larger for IMM (upper panel) than for CPPLS (lower panel), and especially the Negatives (blue curves) seem to stretch into the Positive side, producing false positives. Another issue is that a multivariate approach makes simultaneous use of all the available frequencies and their covariance structure. By taking this into consideration, multivariate analysis can identify important frequency effects and detect contributions from frequencies that are too small to be detected by the univariate Markov chain models. CPPLS will therefore provide superior statistical power compared to the Markov chain models as long as a model selection procedure preventing under- or over-fitting is implemented.

Although CPPLS based on codon frequencies, performs extremely well for ORF classification there are a few Positives missed. In the genome of *Sulfolobus islandicus* we miss an iron-sulfur binding domain protein and some hypothetical proteins. In *Pseudomonas putida* we fail to detect the genes annotated as “RND family efflux transporter MFP subunit”, “copper resistance B”, as well as some hypothetical proteins. In *Mycobacterium tuberculosis* we miss some hypothetical proteins and a “transmembrane serine” protein. For *Escherichia coli* we fail to classify an “intimin adherence” protein as Positive. This is a protein with no clear function defined also found in some *Shigella* and *Citrobacter* species.

We note that these genes are all involved in pathogenecity, e.g. the intimin gene is usually found on pathogenicity islands known collectively as LEE’s [36]. Pathogenicity is a trait prone to be horizontally transferred [37,38]. The fact that these genes are quite different in codon composition from all other HCO’s in their respective populations may indeed be taken as an indication of recent horizontal gene transfer. This illustrates another potential use of coding sequence modeling besides gene finding. When a highly conserved ORF is not recognized as such, it is an indicator of ’foreign’ DNA. The recognition of horizontally transferred genes, which are often linked to virulence factors and antibiotic resistance [39,40], can be aided by the capability of coding sequence modeling. For instance, it is known that the GC content of the third codon position is highly correlated with genomic GC content [41]. Since genomic GC content is associated with the environment of the bacteria [42,43], the codon frequencies of horizontally transferred DNA may be very different to that of the host [43].

## Conclusions

Results of comprehensive comparisons in coding sequence modeling on multiple data sets show that the CPPLS approach provides superior performance compared to the IMM. Furthermore, codon representations were found to be superior in classifying ORFs compared to DNA and protein representations for the CPPLS method. We therefore conclude that a multivariate approach like CPPLS should be more utilized in coding sequence modeling, as well as in pattern recognition problems where sequences are to be classified by their content, like for instance, in the detection of horizontally transferred DNA.

## Competing interests

The authors declare that they have no competing interests.

## Authors contributions

TM and LS initiated the project and the ideas. All authors have been involved in the later development of the approach and the final algorithm. TM has done the programming, with some assistance from SS. TM, ABK, JB and LS has drafted the manuscript, with inputs from all other authors. All authors have read and approved the final manuscript.

## Supplementary Material

Additional file 1 Figure S1. The number of positives against different thresholds. The number of Positive genes obtained for different thresholds *t* for all species. A threshold of *t* = 0.3 means members in a gene cluster differ by no more than roughly 30%, and the ’center’ gene (medoide) in each cluster is used as a Positive. If a species has sequences more than 400, then a sample of size 400 sequences are taken as positives. A small threshold (close to 0) gives fewer, but tighter, clusters. Click here for file
